# Supplementary material for: Most Small Cerebral Cortical Veins Demonstrate Significant Flow Pulsatility: A Human Phase Contrast MRI Study at 7T
Source: Front Neurosci. 2020 May 5;14:415. doi: 10.3389/fnins.2020.00415 (PMC7214844; doi:10.3389/fnins.2020.00415)
Supplement: Supplementary file 1 [file Data_Sheet_1.PDF]

Supplementary Material

1 Supplementary Figures

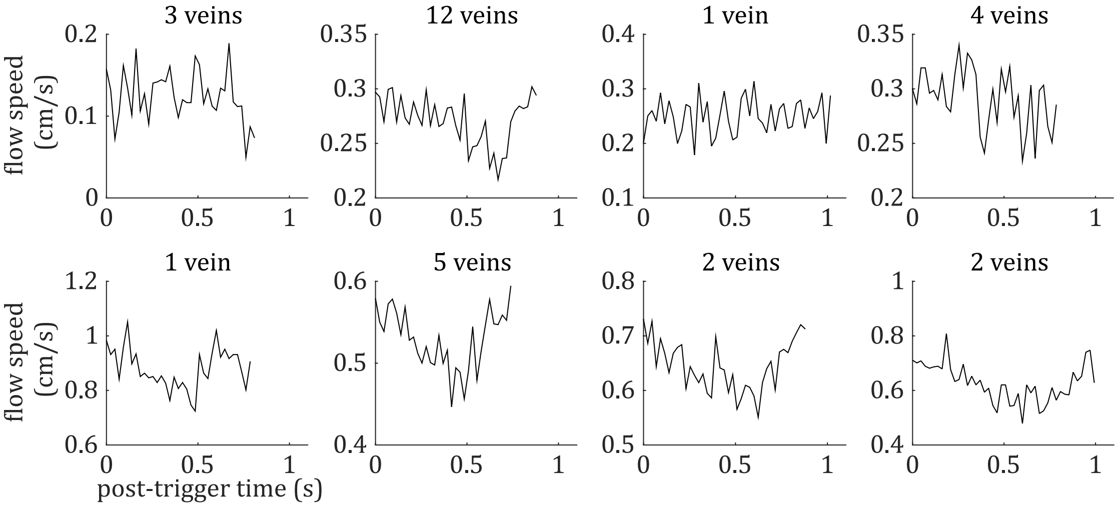

Supplementary Figure S1. Cardiac cycle synchronized venous blood flow time-courses for each subject for veins that do not meet the PCNR>3.9 threshold (i.e. 'non-pulsatile veins'). The number of non-pulsatile veins for each subject is displayed above the respective plot.

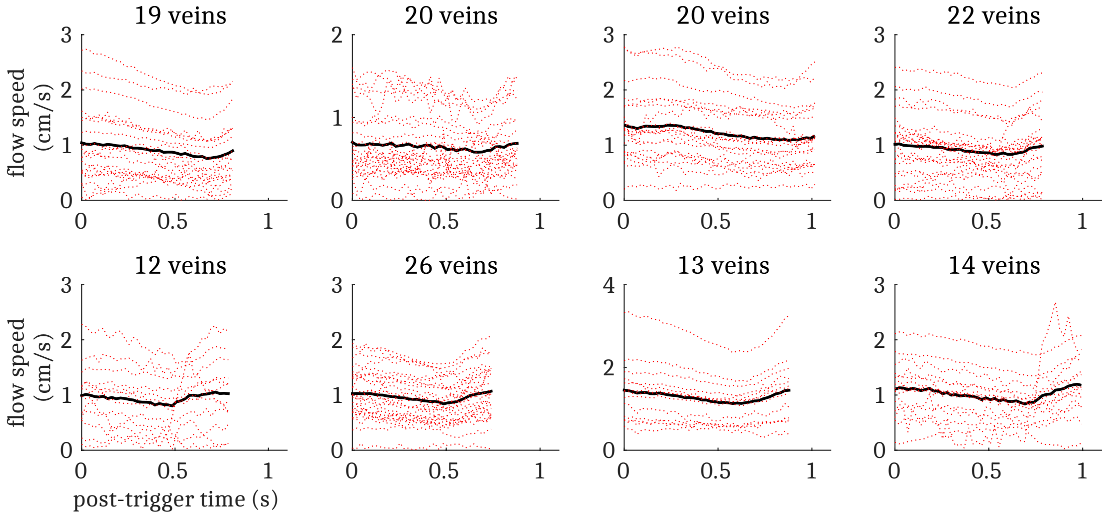

Supplementary Figure S2. Cardiac cycle synchronized venous blood flow time-courses for each subject for every vein (red dotted lines) and for the mean across veins (black line). The total number of veins studied for each subject is displayed above the respective plot.

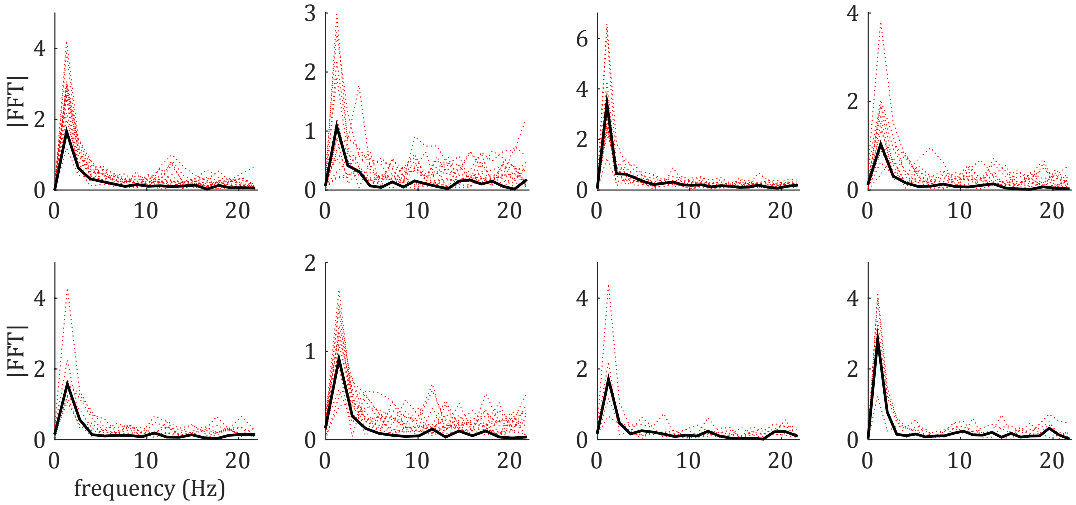

Supplementary Figure S3. Frequency spectra for each subject, for every small vein (red dotted lines) and for the mean across small veins (black line). This was calculated by fast Fourier transform of the time-course. Note that the main peak at ~ 1Hz corresponds to the principle frequency of the cardiac cycle.
